# Small molecules as ligands in the tuning of immune regulatory receptors

**DOI:** 10.3389/fimmu.2025.1648691

**Published:** 2025-08-29

**Authors:** Laura Fioretto, Marcello Ziaco, Genoveffa Nuzzo, Federica Albiani, Marisa Saponaro, Dalila Carbone, Giusi Barra, Mario dell’Isola, Olimpia Follero, Nunzia Esercizio, Daniela Castiglia, Giuliana d’Ippolito, Carmela Gallo, Angelo Fontana, Emiliano Manzo

**Affiliations:** ^1^ Bio-Organic Chemistry Unit, Institute of Biomolecular Chemistry, Consiglio Nazionale delle Ricerche, Naples, Italy; ^2^ National Research Council of Italy, Institute of Biomolecular Chemistry (CNR–ICB), Catania, Italy; ^3^ Laboratory of Bio-Organic Chemistry and Chemical Biology, Department of Biology, University of Naples “Federico II”, Naples, Italy

**Keywords:** small ligands, C-type lectin receptor, immunoglobulin receptors, innate immune system, immunology

## Abstract

Professional antigen-presenting cells (APCs) represent a crucial link between the innate and the adaptive immune response. APCs express specific surface receptors which are primarily involved in “non-self” and/or “self” ligand recognition. Upon ligand binding, these receptors can trigger cell signalling leading to the production of pro-inflammatory cytokines, chemokines and Type 1 interferons, supporting antimicrobial and inflammatory responses. Recently, two major families of receptors, C-type lectin receptors and immunoglobulin receptors, are emerging as potential therapeutic targets to activate and modulate immune system through different intracellular signalling motifs upon binding with endogenous and exogenous ligands. The chemical characterization of the molecular determinants necessary for the receptors/ligands binding promotes the design and optimization of small molecules crucial for the comprehension of biological functions and for the therapeutic treatment of specific receptor-associated disorders. This review focuses on the description of these ligands together with their biological evaluation and their impact on the modulation of the immune response.

## 1 Introduction

The innate immune response constitutes the first barrier against various pathogens. Along with the immediate action of cytotoxic cells, such as natural killer (NK) cells and granulocytes, a more complex response is provided by antigen-presenting cells (APCs), able to initiate the processes pivotal for the antigen-specific adaptive immunity.

Antigen recognition is mediated by receptors families known as pattern recognition receptors (PRRs), manly located at the surface of innate immune cells such as macrophages, dendritic cells (DCs), neutrophils, granulocytes, mast cells, monocytes, basophils, natural killer cells, and epithelial cells (1–3).

PRRs are uptake receptors capable of recognizing molecules associated with pathogens (Pathogen-Associated Molecular Patterns, PAMPs) or released by damaged cells (Damage-Associated Molecular Patterns, DAMPs), to allow their internalization and subsequent processing and presentation to T cells (4).

PRRs are broadly categorized based on their cellular location, structures and functions in different sub-families including Toll-like receptors (TLRs), nucleotide-binding oligomerization domain-like receptors (NLRs), retinoic acid-inducible gene-I-like receptors (RLRs), AIM2-like receptor (ALR) and C-type lectin receptors (CLRs) (5).

In particular, CLRs recognize pathogen-derived ligands, but also natural endogenous ligands such as self-carbohydrates, proteins, or lipids, with implications in the control of tissue damage, autoimmune diseases and tumorigenesis (6, 7).

Circumstantial evidence indicates that DAMPs and PAMPs could also bind immunoglobulin-like receptors such as the triggering receptor expressed on myeloid cells (TREM-1 and TREM-2) (8),. even though they are not yet fully identified as PRRs. Given their tissue and cell-specific expression, there is considerable evidence that immunoglobulin-like receptors could be pivotally implied in modulation of the innate immune response, intracellular signal transduction, and interactions with other signalling cascades, as well as those involving TLRs (9, 10).

Recently, the interest to study and understand the mechanism of either CLRs and immunoglobulin-like receptors families has been growing, given their ability to positively and/or negatively regulate immune cell activation following interaction with a variety of endogenous and exogenous ligands (7, 11). The identification of chemical characteristics of these ligands could improve the understanding of the intracellular downstream signalling and facilitate the study of their physiological role and development in the research of new vaccines or therapeutic treatments. In addition, identification of the endogenous ligands can accelerate the synthesis and optimization of organic compounds, resulting in potentially higher efficiency for further developments.

This review will focus on the functional mechanisms of the most studied CLRs and immunoglobulin receptors, particularly in relation to their interaction with specific ligands and the possible cross-talk among different immune receptors. Special attention will be given to the recognition of small molecules.

**Figure 1:**
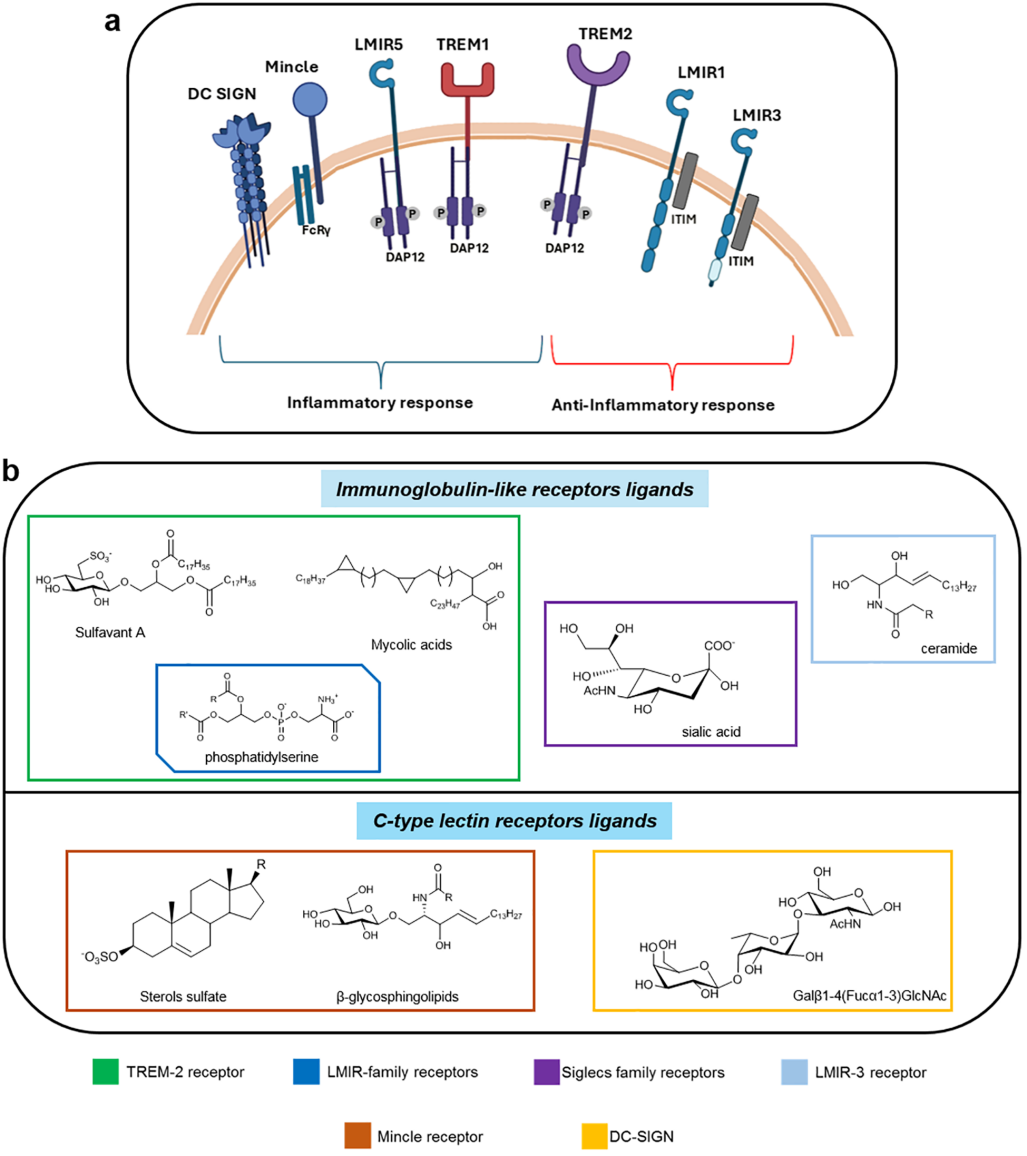
**(a)** Summary of the main classes of immunoglobulin like receptors and CLRs recognizing small molecules and their immunological response. Receptors are represented based on their biological ability to induce inflammatory or anti-inflammatory responses. For TREM-1 and TREM-2 the evaluation is more complex. Both TREM-2 can sustain the cell energetic by the activation of mTOR pathway. (Created with online software BioRender.com); **(b)** Small ligands and ligands structural motifs of immunoglobulin-like and C-type lectin receptors.

Small molecules, characterized by a molecular weight approximately less than 1000 dalton and often of lipophilic nature, are generally easily identifiable, synthesizable, chemically modifiable and potentially suitable for the development of new drugs due to their ability to selectively interact with specific biological targets. (**Figure 1**).

Previous studies on amphiphilic drugs have, in fact, highlighted how specific structural features of small ligands can profoundly influence interaction with immune receptors, providing a rationale for the design of novel immunomodulatory molecules (12).

## 2 Receptors families

### 2.1 Immunoglobulin-like receptors

Immunoglubulin-like receptors are a family of receptors possessing extracellular immunoglobulin domains. They are known to exert immunomodulatory effects on a wide range of immune cells. Based on their cellular-specific expression, they can be classified as leukocyte mono-immunoglobulin-like receptors (LMIRs), triggering receptors expressed on myeloid cells (TREMs), Signal regulatory proteins (SIRPs) or sialic acid binding Ig-like lectins (SIGLECs). These receptors can have both activating and deactivating immunological characteristics.

**Figure 2:**
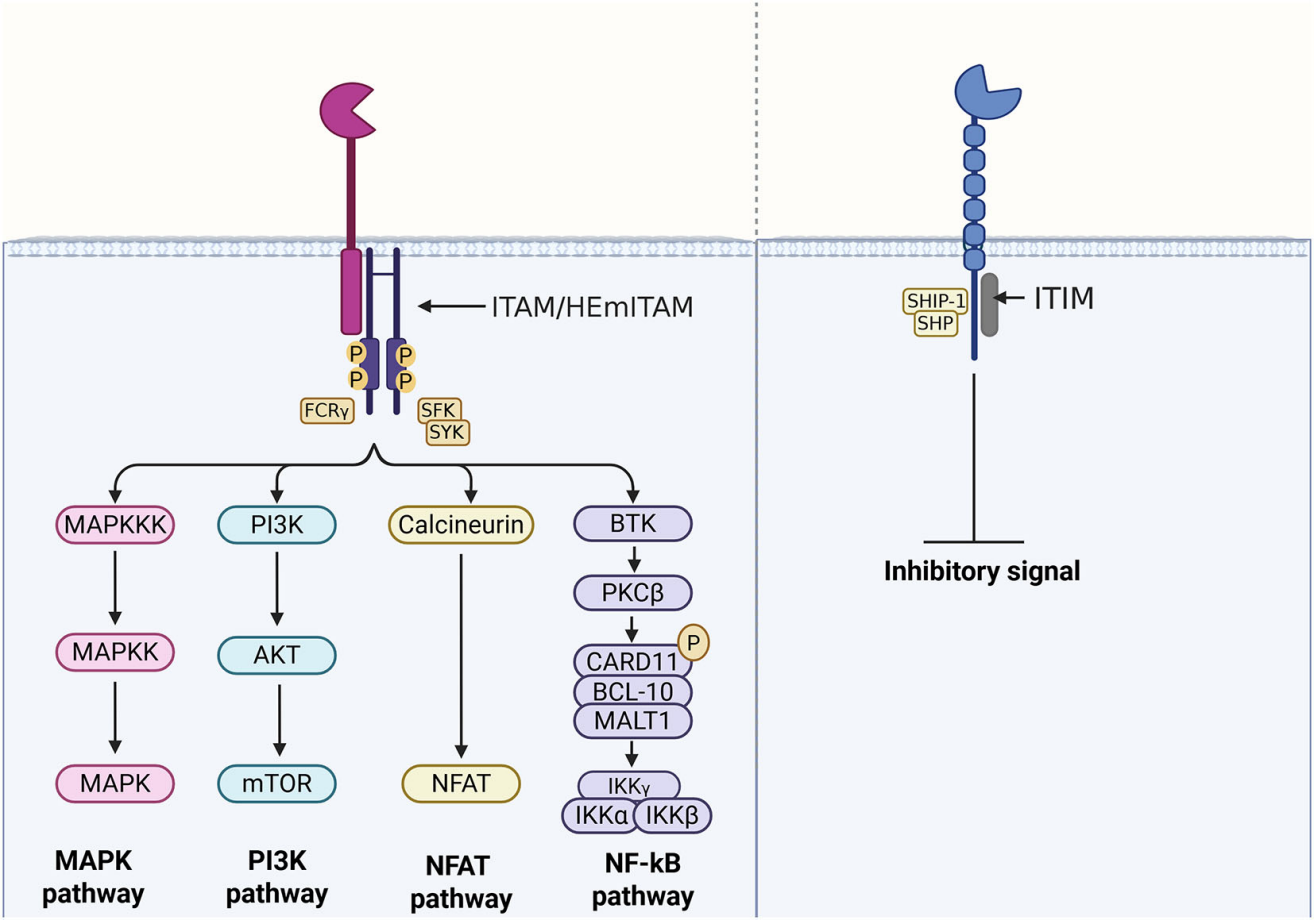
Representation of ITAM- and ITIM-mediated activation and inhibition pathways.

Activating receptors generally associate with an immunoreceptor tyrosine-based activation motif (ITAM or hemITAM) or a transmembrane adaptor protein containing a related activation motif, such as DNAX-activating Protein 10 (DAP10), DNAX-activating Protein 12 (DAP12), or the Fc-gamma receptor (FcRγ). DAP10, DAP12, and FcRγ act as substrates and docking sites for kinases, enabling the amplification of intracellular signalling reactions (13). In contrast, inhibitory receptors contain an immunoreceptor tyrosine-based inhibitory motif (ITIM) in their cytoplasmic domain (14–17), which facilitates the recruitment of phosphatases (**Figure 2**). In this regard, ‘self’ or ‘non-self’ molecules, able to interact and activate these receptors, enclose the enormous potential for controlling and modulating the immune response and the resulting cellular homeostasis.

Although in fact, some receptors display distinct specificity profiles, they may exhibit overlapping ligand-binding patterns (**Table 1**).

**Table 1:** Overview of ligands of Immunoglobulin-like receptors.

Receptor	Ligand	Biological function	Reference
LMIR-1	Phosphatidylserine	Apoptosis	(24, 31)
	Phosphatidylethanolamine		
LMIR-2	Unknown	–	–
LMIR-3	Ceramide, sphingomyelin	Activation of mast cell	(41)
	Sphingosylphosphorylcholine	Activation of mouse bone-marrow-derived mast cells (BMMCs)	(38)
	Phosphatidylserine	Apoptosis	(42)
LMIR-4	Unknown, maybe same as LMIR-3	–	(34)
LMIR-5	TIM1 bound to phosphatidylserine	Cytokine production by peritoneal macrophage	(48)
	Phosphatidylserine	–	(43)
	Phosphatidylethanolamine		
	Phosphorylcholine		
	Glycolipids GD1a, GQ1b, and crude gangliosides	–	(52)
	3-O-sulfo-D-galactosylceramide	NFAT activation pathway; MCP-1 production, but not the production of proinflammatory cytokines	(52)
	Glycoconjugates	–	(53)
LMIR-7	Unknown	–	–
TREM-1	High Mobility Group Box 1	Activation of inflammatory response	(87, 89)
	Heat Shock Protein 70	Activation of TNFα and IFNγ mRNAs expression in monocytes and of IL-2 secretion by PBMCs	(88)
	Peptidoglycan recognition protein 1	–	(91)
TREM-2	Dextran sulfate, LPS, and LTA	–	(110)
	Lipoligosaccharides (LOS) from *N. Gonococcusan*	Interleukin-6 production in HeLa cervical carcinoma cells	(113)
	unknown ligand in *C. jejuni*	–	(113)
	Phosphatidylserine	Sustaining of the microglial response to Aβ accumulation	(43, 103)
	Phosphatidylethanolamine		
	Cardiolipin		
	Nucleic acids released by ischemic brain	Microglial activation to amoeboid phenotype and increase phagocytosis of injured neurons in brain ischemia	(115)
	*Escherichia coli*	–	(112)
	Apolipoprotein	Increase of phagocytosis in microglia	(111)
	Aminophospholipids	Regulation of microglial function by transducing intracellular signals on apoptotic cells	(115)
	Beta amyloid forms	Activation of NFAT signaling and internalization of beta amyloid forms	(114)
	Glycolipids and sulfoglycolipids	–	(52, 117, 126)
	Non-glycosylated mycolic acid-containing lipids	–	(118, 119)
	Sphingosine-1-phosphate	–	(120)
SIRP-α	CD47	Downregulation of innate and adaptive anti-tumor immune response	(141)
	surfactant proteins A and D	Stimulation of anti-inflammatory response in macrophages	(144)
SIRP-β	Unknown	Activation of phagocytosis in macrophages	(140)
SIRP-γ	CD47–Interaction 10 times weaker compared to SIRP-α	Downregulation of innate and adaptive anti-tumor immune response	(142)
Siglecs	Sialic acids or sialylated structures of HIV- Porcine reproductive and respiratory syndrome virus–tumors	Immune response suppression in favour of pathogen-tumor survival	(164–166)

#### 2.1.1 LMIR/CD300 family members

The LMIR (also called CD300) (18, 19) family belongs to the paired immune receptors. Lipids or lipid-binding proteins have been identified as ligands for several CD300/LMIR members. Despite the similarity in the extracellular Ig-like domains, there are relevant structural differences between activating and inhibitory receptors, pivotal for the fine tuning of immune response. Therefore, we can distinguish them into two main groups: inhibiting receptors, LMIR-1 and LMIR-3, and activating receptors, LMIR-2, LMIR-4, LMIR-5 and LMIR-7.

##### 2.1.1.1 Inhibitory LMIR receptors

###### 2.1.1.1.1 LMIR-1/CML8/CD300a

LMIR-1 (also called CML8 or CD300a) is an inhibitory receptor, containing several ITIM motifs in the cytoplasmic domain (10, 19). LMIR-1 is expressed in myeloid and lymphoid cells, particularly in mast cells, eosinophils, and basophils, the three most important cell types involved in the initiation and regulation of allergic responses. Indeed, evidence indicates that inhibition of LMIR-1 induces mast cell degranulation by SCF in a murine model of cutaneous anaphylaxis (20). Targeting LMIR-1 was shown to inhibit, through the regulation of eosinophil and mast cell signalling *in vivo*, the bronchoalveolar lavage fluid inflammation, lung remodelling and inflammation in a model of chronic established asthma (21).

Phosphorylated ITIM motifs are able to recruit different phosphatases depending on the cell type where LMIR-1 is expressed (22). It was demonstrated that the corresponding LMIR-1 in humans, when cross-linked, inhibits IgE-induced degranulation and SCF-mediated survival on mast cells, through a mechanism that involves tyrosine phosphorylation and phosphatase recruitment (20, 23). These results highlight a new role for the regulation of human allergic response through LMIR-1, even though the endogenous ligand involved in such mechanisms must be still revealed.

Interestingly, knockdown of LMIR-1 in a mice model of acute septic peritonitis showed prolonged survival, given the greater expression of chemoattractants in peritoneal mast cells, leading to increased neutrophil recruitment and a better bacterial clearance (24). This evidence indicated a new regulatory role of LMIR-1 for mast cell inflammatory responses to microbial infections. LMIR-1 was also identified as a new marker for acute lymphoblastic leukemia, as differentially expressed compared to the wild type (25). In HIV infection, LMIR-1 is deregulated on B cells, suggesting the possibility that this receptor may contribute to the B-cell dysfunction observed in HIV-infected patients (26). Moreover, LMIR-1 has been associated with susceptibility to psoriasis (27).

Lack of LMIR-1 increases the secretion of pro-inflammatory cytokines produced by TLR4/MyD88 in macrophages and impaired wound healing (28, 29). LMIR-1 is able to bind phospholipid molecules as phosphatidylserine (PS) and phosphatidylethanolamine (PE), exposed as ‘eat me’ signals on the outer leaflet of the plasma membrane of apoptotic cells, forming cavity for the ligands polar heads insertion (30, 31). More selectively, human chimeric LMIR-1 exhibits a binding stronger to PE than PS, modulating the ingestion of dead cells (31).

Interestingly, considering that aluminum salts (alum) have been widely used as vaccine adjuvant, it was demonstrated that LMIR-1 expression was upregulated on inflammatory DCs after injecting mice with alum, and involved in the generation of dead cells in the peritoneal cavity. In this regard, inflammatory DCs bound dead cells via LMIR-1/PS interaction, resulting in Th2 lymphocytes responses and enhanced allergic airway inflammation, thus suggesting the involvement of LMIR-1 in alum-induced Th2 skewing (32). Furthermore, blocking LMIR-1/PS interaction may have therapeutic applications for the prevention or treatment of vaccine-involved pathologic conditions and allergic airway inflammation (32).

These studies demonstrated a novel pathway of cell regulation which modulate allergic responses and microbial infections, indicating LMIR-1 as a candidate target. Therefore, further investigation aimed at discovering possible antagonists for LMIR-1 will be useful for future treatment or for understanding the underlying molecular mechanism of associated diseases.

###### 2.1.1.1.2 LMIR-3/CLM1/CD300f

The receptor LMIR-3 (also called CD300f or CLM1) is another inhibitory receptor which delivers its inhibitory signal via two ITIMs and a single immunoreceptor tyrosine-based switch motif (ITSM) that can recruit Src homology 2 domain-containing protein phosphatase-1 (SHP-1) and/or SHP-2 (33). LMIR-3 is highly expressed in myeloid cells, particularly mast cells (34). Interestingly, LMIR-3 is weakly expressed in monocyte-derived DCs, but is strongly upregulated when cultured in the presence of 1,25-dihydroxyvitamin D3, which reprograms DCs toward a tolerogenic phenotype, suggesting an important role for this receptor in the maintenance of immune tolerance (35).

LMIR-3 has been shown to have a neuroprotective role in a rat model of acute brain injury (36). Indeed, LMIR-3 is a negative regulator of myeloid effector cells in autoimmune demyelination, therefore implied in multiple sclerosis and autoimmune encephalomyelitis (37). Furthermore, LMIR-3 plays a pivotal role in regulating the mast cell-dependent allergic responses in mice (38).

Downregulation of LMIR-3 in DCs enhances T cell proliferation initiated by DCs, as well as antigen-specific T cell responses, both *in vitro* and *in vivo*, leading to effective protection against tumor challenge in mice (39). Block of LMIR-3 significantly reduced the engraftment of primary human acute myeloid leukemia cells, highlighting the potential LMIR-3 in tumor immunotherapy (40).

As possible ligands, several extracellular lipids including ceramide, sphingomyelin and sphingosylphosphorylcholine (SPC) were found (38, 41). The binding with these lipids results into an inhibition of the high-affinity IgE receptor (FcϵRI)-mediated activation of mouse bone-marrow-derived mast cells (BMMCs) (38). Particularly, it was evident that the ceramide-LMIR-3 interaction was pivotal in LMIR-3-mediated inhibition of mast cell activation *in vivo*, through a not fully understood mechanism. Indeed, a deeper examination is required to comprehend how LMIR-3 could influence FcϵRI signalling *via* colocalization of ceramide lipids and FcϵRI itself in mast cells. The influence of ceramide is supported by different research groups, but there is contrasting evidence about the binding affinity of other lipids proposed as possible ligands. Choi proposed PS and PE to be potential ligands for chimeric LMIR-3, resulting in increased phagocytosis (42). However, given that both PS and PE were expressed on dead cells, it was unclear how an inhibitory receptor as LMIR-3 could promote phagocytosis. These observations of Choi were not confirmed by Izawa, who showed that LMIR-3 on mast cells only bound membrane immobilized ceramide, but not other phospholipids as PS, PE, or phosphatidylcholine (PC) (38). The latter, however, appeared to be a ligand for LMIR-3 in the reporter cells but not in mast cells (38), possibly due to slight structural difference of recognition domain between endogenous LMIR3 and the chimera receptor LMIR3-CD3ζ used in reporter cell assay and/or for possible surface components-induced interference of PC-LMIR3 interaction on mast cells. Therefore, with the aim to identify other possible relevant ligands for LMIR-3, more in depth studies and analysis should be performed.

Notably, both LMIR-1 and LMIR-3 can bind to dead cells in a calcium dependent manner, suggesting that these two receptors must interact with evolutionary conserved ligands (10).

Circumstantial evidence suggests possible similarities among LMIR-1, LMIR-3 and the immunoglobulin-like receptor TREM-2 due to the high conservation of their domain architecture and the structural motifs, that could allow the binding with similar ligands or classes of ligands (e.g. PS and PE) (43).

##### 2.1.1.2 Activating LMIR receptors

###### 2.1.1.2.1 LMIR-2/CLM4/CD300C

The receptor LMIR-2 (also called CD300C or CLM4) is an activating receptor which is mainly expressed on macrophages and a subset of B cells in the spleen and the peritoneal cavity (19). LMIR-2 mediates an activating signal through the association with FcRγ or DAP12, for the production of the proinflammatory cytokines tumour necrosis factor (TNF)-α and interleukin (IL)-6 in macrophages (44). LMIR-2 regulates TLR4-mediated cell adhesion to VCAM-1 in highly purified inflammatory monocytes in response to TLR ligands alone (45). These data suggest that a ligand for LMIR-2 could be expressed after TLR ligand stimulation, resulting in cis-binding between LMIR-2 and the unknown ligand. Even though its physiological role is not fully understood, Totsuka et al. suggested that activation of LMIR-2 by TLR4/MyD88-mediated signalling is essential for the transmigration of inflammatory monocytes from the blood to sites of infection puncture (CLP)-induced peritonitis. The authors proposed that LMIR-2 may be able to bind phospholipids as functional ligands, similarly to the other CD300 family members and the TREM family proteins (45). As phospholipids are dynamically remodelled on stimulation with innate stimuli such as TLR ligands (46), its activation depends on TRL4/MyD88 axis. However, unlike LMIR-1, LMIR-2 does not bind PS (24).

Further efforts and studies are in progress to identify possible ligands that could potentially interact with LMIR-2 and unravel its function *in vivo*. Moreover, it will be interesting to discover if other ligands, able to interact with LMIR-1 or TREM-2, besides PS, could potentially bind also LMIR-2.

###### 2.1.1.2.2 LMIR-4/CLM5/CD300ld

LMIR-4 (also called CD300ld or CLM5) is a further activating receptor which is physically associates with ITAM-bearing adaptor FcRγ. LMIR-4 is preferentially expressed on neutrophils in the peripheral blood, bone marrow, peritoneal cavity and spleen (47). LMIR-4 is considered a counterpart of the inhibitory receptor LMIR-3 (34). It has been shown that intraperitoneal administration of the TLR4 agonist lipopolysaccharide (LPS) strikingly up-regulates LMIR-3 and down-regulates LMIR-4, whereas granulocyte colony-stimulating factor up-regulates both LMIR-3 and LMIR-4 in granulocytes. These results suggest that innate immune system is partially regulated by the qualitative and quantitative balance of the paired receptors LMIR-3 and LMIR-4 (34). However, truly little is known about LMIR-4 function in immune disease. We could speculate that, as counterpart receptor of LMIR-3, LMIR-4 could be involved in similar mechanisms pathways of LMIR-3.

LMIR-4 synergistically enhanced TLR4 signalling in mast cells and granulocytes, allowing a robust cytokine production in accordance with enhanced activation of ERK. Upon binding with unknown ligand, LMIR-4 could positively regulate various signalling pathways, affecting the inflammatory responses of myeloid cells (34).

Taking into consideration that LMIR-3 and LMIR-4 share high homology in the Ig-like domain, these receptors may share the same ligands, with consequence that these molecules could be able to engage simultaneously the two receptors, mimicking cell physiological conditions (34). Moreover, their identification is pivotal for any further understanding of LMIR-3 and LMIR-4 functions as they could represent a fine balance between the inhibiting-activating mechanisms. Elucidation of the role of LMIR-4 could unravel its contribution to inflammatory processes,crucial for future strategic therapies.

###### 2.1.1.2.3 LMIR-5/CLM7/CD300lb

LMIR-5 (also called CD300lb or CLM7) is an activating receptor coupled to DAP-12. LMIR-5 is mainly expressed in myeloid cells, such as neutrophils, peripheral macrophages, and mast cells (10).

LMIR-5 can interact with T cell Ig mucin 1 (TIM1) but similarly also with TIM4. Interestingly, the binding site of LMIR-5 to TIM1 is located in proximity of TIM1 PS-binding site, which *per se* is not able to bind PS (48). These results are in contradiction with previous reports which showed LMIR-5 able to independently bind to PS, PE, and PC (43). Importantly, soluble form of LMIR-5 (sLMIR-5) upon binding to an unidentified ligand, other than TIM-1 and TIM-4, is involved in induced cytokine production by peritoneal macrophage (49). LMIR-5 deficiency profoundly reduced systemic cytokine production and septic mortality in LPS-administered mice, pointing out a peculiar relation with the triggering receptor expressed on myeloid cells-1 (TREM-1), an inflammatory receptor (49). Indeed, stimulation with TLR agonists increased the release of both soluble forms sLMIR-5 and sTREM-1. Although sTREM-1 attenuates excessive inflammatory response by counter-regulating TREM-1, inflammatory amplifier in sepsis, sLMIR-5 amplifies LPS-induced lethal inflammation (50, 51). The relation with TREM-1 is pivotal for further comprehension of its role, as well as the discovery of new ligands possibly shared by LMIR-5 and TREM-1, also justified by the strong similarities of the LMIR and TREM families (43).

LMIR-5 interacts with glycolipids GD1a, GQ1b, and crude gangliosides (52). In particular, LMIR-5 can bind with highest affinity to 3-*O*-sulfo-β-D-galactosylceramide C24:1, recognizing the 3-*O*-sulfo-D-galactose moiety, which resulted in DAP12-mediated NFAT activation (53). It was shown that the bacterium *C. jejuni* activates NFAT through DAP12 by interaction with LMIR-5, whose activators were identified as protein components, RNA-associated proteins, and 150-kDa high-molecular-weight glycoconjugates (53). However, the identities of these LMIR-5 activators should be further investigated.

LMIR-5 plays a key role in increasing acute kidney damage, characterized by tubular necrosis and cast formation. Indeed, LMIR-5, as described above, strongly binds to TIM1 (48), which was identified as both marker of acute kidney injury (54) and marker in renal carcinoma and generally associated with immune dysfunction. Therefore, hampering LMIR-5-TIM1 interaction might be a novel therapeutic strategy for acute renal tubular damage (48).

LMIR-5 is one of the most well studied LMIR receptors from the ligand binding prospective. However, most of its ligands are still unknown, and their discovery is crucial for further investigation of the role in the activation of immune response and in macrophage activation. Given the strong similarity that LMIR-5 shares with TREM-1 as an inflammatory amplifier, it may be interesting to study the similarities of activation pathways and possible shared ligands. Peculiarly, both receptors present soluble forms similarly formed under TLR stimulations, but with opposite functions. Therefore, the fine control and balance between soluble and transmembrane forms of TREM-1 and LMIR-5 seems to be extremely important to sort out the complex ways of immunomodulatory regulation.

###### 2.1.1.2.4 LMIR-7/CLM3/CD300lh

LMIR-7 (also called CD300lh or CLM3) is an activating receptor highly expressed in mast cells, monocytes and macrophages (55). LMIR-7 synergizes with TLR4 in signalling and binds to FcRγ, but with lower affinity compared with LMIR-4-FcRγ (55). It has been shown that LMIR-7 functions as a positive regulator of TLR9 (56). LMIR-7 upregulates TLR9-mediated production of the proinflammatory cytokines TNF-α and IL-6 but does not affect type I-IFN expression (56).

Interestingly, LMIR-7 shares high homology similarity with LMIR-4 in the amino acid sequences of Ig-like and transmembrane domain (55). Therefore, it is possible that LMIR-7, as well as LMIR-4, modulates the innate immune responses in a cell type-dependent manner. Overall, with the aim to improve the understanding of *in vivo* functions of LMIR receptors, both analysis of the knock-out mice and identification of the ligands for each LMIR are necessary (55).

Considering that LMIR-4 is a counterpart of inhibitory receptor LMIR-3 and that LMIR-7 shares a high degree of homology with LMIR-4, it is crucial to better understand the peculiar function of LMIR-7 in relation to LMIR-4 and understand its possible analogous role to LMIR-3. The LMIR-3-LMIR-4-LMIR-7 axis could be, potentially, a new immunomodulatory regulation mechanism that needs to be further explored.

#### 2.1.2 TREM family members

TREM receptors are a class of cell surface receptors characterized by a single V-type immunoglobulin domain in their extracellular region (57).

TREM family members are expressed by granulocytes, monocytes and tissue macrophages. These receptors participate in diverse cell processes, including inflammation, bone homeostasis, neurological development and coagulation (58). In this family, TREM1 and TREM2 are the best characterized proteins. The growing interest for the elucidation of the cell signalling pathways of these receptors with high therapeutic potential, raised the focus on the identification of endogenous TREM ligands.

##### 2.1.2.1 TREM-1 and TREM-2 receptors

TREM-1 and 2 proteins are structurally related, consisting in a single extracellular variable-type immunoglobulin (Ig) domain; a shorter stalk region; a transmembrane domain and a short cytoplasmic tail not containing any activation, signalling or trafficking motifs (59).

The transmembrane domain includes a charged lysine residue that enables the interaction with DAP12 (58). Upon binding with DAP12, TREM receptors can potentially have activating and/or inhibitory roles in innate immune responses. Although this hypothesis remains highly speculative, TREM-1 and -2 proteins show different and specialized functional roles, especially in myeloid cells (60).

TREM-1 and TREM-2 extracellular domains can also be found in a soluble forms (sTREM-1 and sTREM-2), which can be released by a proteolytic cut within the protein stalk region (61) or by alternative splicing (62). Due their intrinsic structural characteristics, TREM-1 and TREM-2 can act in a dependent manner *via* its interactor protein by which initiate intracellular signalling or in independent manner as a soluble protein.

The involvement of these receptors in inflammation, neurodegenerative diseases and in cancer is widely recognized and further studies are pivotal for the definition of their biology (57).

###### 2.1.2.1.1 TREM-1 receptor

TREM-1 is mainly expressed on myeloid cells such as monocytes, macrophages and granulocytes (63), but is also detected on parenchymal cell types such as bronchial, corneal, gastric epithelial cells, and hepatic endothelial cells (64–66). TREM-1 expression is upregulated during septic shock, in a number of infectious diseases, such as pneumonia (67) and suppurated cholangitis (68), in obstructive nephropathies (69) and chronic kidney diseases (70). TREM-1 can be also upregulated on macrophages in lung cancer with subsequent inflammatory response, inducing complications and death (71). Furthermore, TREM-1 plays a pivotal role in cardiovascular diseases, such as atherosclerosis pathogenesis (72), acute myocardial infarction (73), coronary artery diseases (74), myocardial dysfunction in septicaemia (75) and infective endocarditis (76). TREM-1 ameliorates neuroinflammatory responses in Parkinson disease, and this neuroprotective effect might occur via the activation of autophagy and anti-inflammatory pathways (77). TREM-1 can promote mitochondrial integrity and cell survival (78, 79), as well as induce rearrangement of the actin cytoskeleton (80), and release of pro-inflammatory cytokines and chemokines, such as MCP-1, MIP1-α, IL-1β, IL-6, IL-8, TNFα (81).

For several years since the discovery of TREM-1 in 2000, no endogenous TREM-1 ligands have been identified. Two studies have proposed peptidoglycan recognition protein 1 as a ligand for TREM1 in *in vitro* assays conducted on PBMCs and linked to the secretion of pro-inflammatory cytokines (82, 83).

The presence of TREM-1 ligand on human platelets could be related to neutrophil activation in the occurrence of microbial stimuli like LPS (84), but also in this case the chemical characterization of the putative implied ligand was unsuccessful.

During the last years, some progress has been made to better understand the role of TREM-1 during chronic tissue damage. It has been reported that extracellular actin co-localizes with TREM-1 in lung tissue sections from septic mice, suggesting that TREM-1 recognizes actin during activation in sepsis (85). Furthermore, actin is considered as TREM-1-interacting protein on platelets (85), although the receptor could be able to recognize other various molecules. High Mobility Group Box 1 (HMGB1) and heat shock proteins, secreted by activated myeloid cells and released by dying and necrotic cells, thus functioning as a DAMP molecule (86–88), were suggested as TREM-1 ligands (89). HMGB1 alone seems unable to induce TREM-1 activation, therefore suggesting the presence of other co-activating molecules (87). In addition, overstimulation of TREM-1 can lead to cell death and release of both actin and HMGB1. This process can provide a large number of ligands for activation of TREM-1 signalling and thus induce some progressive systemic inflammatory response, resulting in sepsis (85).

New findings suggest that the extracellular cold-inducible RNA-binding protein (eCIRP), a recently characterized DAMP, is an endogenous ligand for TREM-1 (90). The binding between eCIRP and TREM-1 can dramatically enhance inflammation during sepsis in macrophages and a peptide-mediated blocking of this interaction significantly improves the outcome, increasing the survival rate (90).

TREM-1 also binds to peptidoglycan recognition receptor 1 (PGLYRP1/Tag7), mainly found in granulocytes and known for its bactericidal properties (91). Interestingly, PGLYRP1 alone was not able to induce TREM-1 activation. In this regard, it is not known whether PGLYRP1 signalling is TREM-1 mediated and if the binding is responsible for the bactericidal properties (87). Recently, a deep characterization of the multifunctional protein PGLYRP1 led to the identification of the peptide (called N3) responsible for the interaction with TREM-1. The N3 peptide corresponds to the *N*-terminal 24 amino acids domain RYVVVSHTAGSSCNTPASCQQQAR (isolated and synthetized). Its interaction with TREM-1 causes the protein dimerization and activation of cytotoxic lymphocytes (83).

TREM-1 does not appear to participate in recognition of lipids, in contrast to other TREM family members (43, 92).

All these results suggest that the identification of an endogenous ligand for TREM-1 is of pivotal importance to fully understand its mechanism of activation and signalling in different inflammatory diseases (87).

In humans, TREM-1 is involved in the amplification of pro-inflammatory innate immune response for the elimination of pathogens (81). The receptor can amplify TLRs response such as TLR4 or TLR2 signalling and synergistically increase the production in pro-inflammatory cytokines such as TNF-α and IL-6 (93). TREM-1 appears to play a crucial role during the initiation of cytokine response in septic shock as well as in other infections (51), through common signalling pathway activation including PI3K, ERK1/2, IRAK1 and Nf-κB (94).

TREM-1 blockade is considered a potential therapeutic procedure, as this strategy could present the peculiar advantage to not fully abolish the inflammatory response required for a proper immune response. For this reason, TREM-1 inhibitors could be safer in treating inflammatory diseases compared to any other immune receptors (87).

###### 2.1.2.1.2 TREM-2 receptor

**Figure 3:**
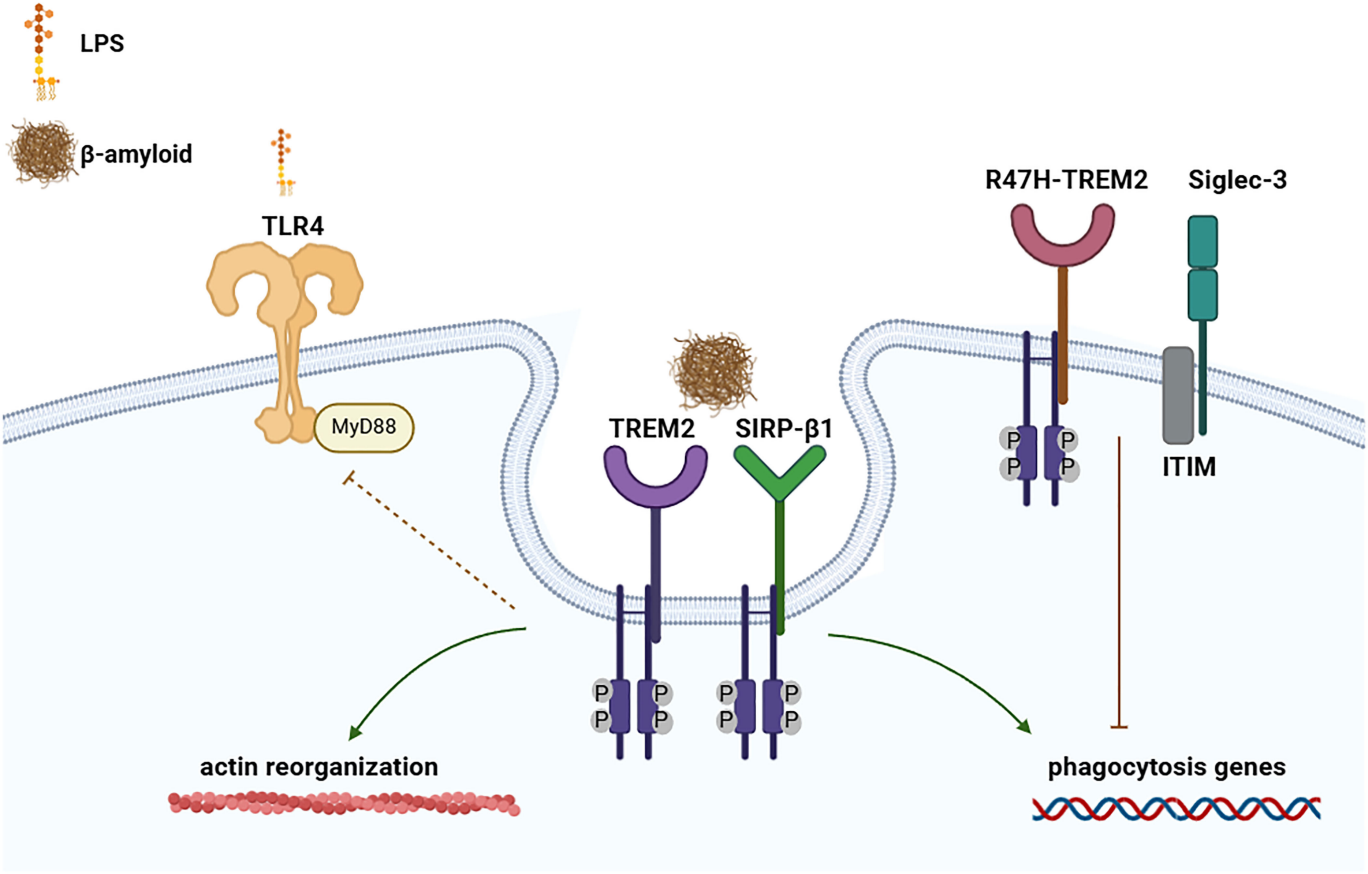
Involvement of TREM-2, SIRP-β1 and Siglec-3 in the development of Alzheimer disease. Representation of TREM-2 and SIRP-β1 in inducing phagocytosis, actin reorganization and reduction of LPS-TLR induced response. Whereas TREM2 genetic modification’s R47H-TREM2, as well as Siglec-3, can inhibit β-amyloid uptake by suppressing the phagocytosis genes. (Created with online software BioRender.com).

TREM-2 is mainly expressed in microglia, in DCs, osteoclasts, Kuppfer cells and alveolar macrophages, and is an important negative regulator of autoimmunity (95, 96). TREM-2 activity or dysfunction are strictly connected to the induction of neurodegenerative disorders. Indeed, recent genetic studies have found that TREM2 mutations represent a significant genetic risk factor in Nasu-Hakola Disease (97), frontotemporal dementia (FTD) (98), Parkinson’s disease (PD) (99), and amyotrophic lateral sclerosis (ALS) (100). Recently, the importance of TREM-2 has been highlighted by the identification of coding variants, such as the TREM2-R47H form, that increase risk for Alzheimer’s disease (**Figure 3**) (101, 102). Also, cytokines released by TREM-2 activation are essential for maintaining microglial metabolic fitness besides enabling microglial activation, migration and phagocytosis, and allowing differentiation into a cell mature profile (103, 104). TREM-2 expression rises during various forms of liver injury in both mice and humans, attenuating TLR4-driven proinflammatory responses (105). TREM-2 is also expressed by adipose tissue macrophages and is extremely important in the regulation of inflammation during obesity. This protein is upregulated in the adipose tissue of dogs on a high-fat diet (HFD) and in the mesenteric adipose tissue of insulin-resistant-diabetic mice, TREM2 promotes adipogenesis by upregulating adipogenic regulators and inhibiting the Wnt10b/β-catenin signaling pathway (106).TREM-2 also sustains cell energetic and biosynthetic metabolism modulating ATP levels and biosynthesis thought the mTOR pathway (107, 108). TREM-2 knockout animal model showed a dramatic reduction of glucose metabolism throughout the brain (109).

The first ligands that have been identified to bind to TREM-2 were bacterial, poly-anionic molecules and anionic bacterial carbohydrates, particularly dextran sulfate, LPS, and LTA (110) and lipid-binding proteins (111). TREM-2 can also directly bind to lipoligosaccharides from *Neisseria gonorrhoeae* and to unknown ligand in *Campylobacter jejuni*, gram-negative bacteria, causative agent for food poisoning (112, 113). Additional studies showed that TREM-2 was able to interact with different beta amyloid forms, increasing their internalization (114), unlike TREM2 missense variants, highlighting the potential crucial role of the protein in the developments of new pharmacological treatment for neurodegenerative pathologies (**Figure 3**) (102).

TREM-2 binds the phospholipids PE, PS, and cardiolipin (CL) (43, 103) as anionic molecules from mammalian cells. In addition to phospholipids, also nucleic acids released by ischemic brain lysate bind to TREM-2. Only cellular fractions containing nuclei or purified DNA, but not cytosolic fractions, were able to induce the signalling through TREM-2 (115). Finally, TREM-2 is able to bind also aminophospholipids that are on apoptotic Neuro2a cells as well as some normal cultured cells (116), glycolipids and sulfoglycolipids (52, 117).

Several studies report the presence of different ligands class of molecules that bind TREM-2, and analogously other immunoglobulin like receptors previously described, as LMIR-1, able to recognize PS and PE. Moreover, those common features further confirm the link between TREM2 and LMIR-1, helping to understand how they could synergise or differ their signalling pathway. More generally, a correlation between TREM and LMIR receptor families was highlighted by Cannon et al (43), suggesting that the TREM/LMIR system may discriminate immunological stimuli based on lipid signatures, thereby influencing downstream responses.

It must be pointed out that LMIR-1 and LMIR-3 are inhibitory receptors which deliver their signal via ITIM domain. Furthermore, TREM-2, similarly to the other activating receptor acts *via* DAP12, but it can deliver inhibitory signals also. These information could highlight a more singular activating/modulating role of TREM-2 in comparison to the other immunoglobulin like receptors.

Considering the importance of TREM-2 and the peculiar similarity with other receptors described in this review, the identification of a unique and specific ligand for this receptor is fundamental.

Recently, interesting advancement concerning the development of new TREM-2 ligands have been done. Lately, non-glycosylated MA-containing lipids of *mycobacteria* were identified as TREM-2 ligands with the activation of TREM-2-DAP12 signalling. In particular, long (C_60_–C_90_) and branched alkyl chains are required for the TREM-2 recognition (118, 119). Moreover, another study reported Sphingosine-1-phosphate (S1P) and an its analogue as endogenous ligand of TREM-2 able to promote phagocytosis (120).

**Figure 4:**
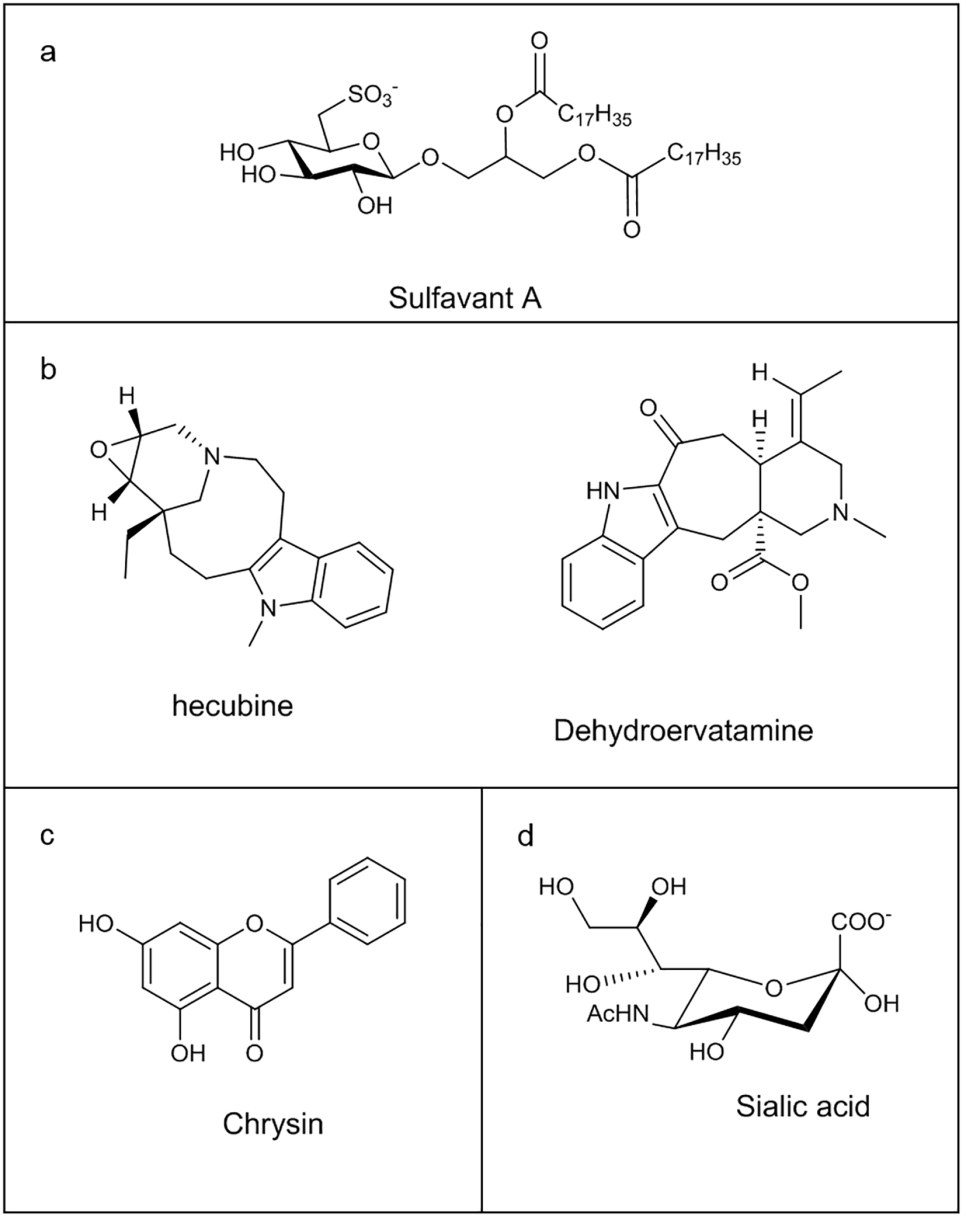
Chemical structure of some Immunoglobulin-like receptors ligands: **(a)** Sulfavant A; **(b)** Hecubine and Dehydroervatamine; **(c)** Chrysin; **(d)** Sialic acid.

A recent crucial progress concerning the essential finding of a small ligand of TREM-2 is represented by Sulfavant A (121, 122), a small nature-inspired sulfo-containing glycolipid with a promising adjuvant property (**Figure 4a**) (123–125). The interaction of Sulfavant A with TREM-2 resulted in a novel cell regulatory function, contributing to immune homeostasis and preserving lymphocyte activation and immune response. Sulfavant A elicited an unconventional hDCs maturation with up-regulation of the costimulatory molecules without production of conventional inflammatory cytokines (117, 126).

Natural monoterpenoid indole alkaloids, hecubine and dehydroervatamine (**Figure 4b**), isolated from *Ervatamia hainanensis* and *Tabernaemontana bovina* plants, respectively, induced neuroinflammation reduction through TREM2 targeting in LPS-stimulated microglia model, preventing pro-inflammatory cytokines release and favouring anti-inflammatory factors expression, with the consequent regulation of the immune response towards a neuroprotective cellular state. *In silico* and cellular thermal shift assay (CETSA) experiments highlighted the direct binding of these molecules with TREM2 (127, 128).

Finally, *Alpinia oxyphylla* fructus extracts, main constituents of some Chinese medicines, have proven to be effective in improving cognitive ability, anti-oxidative stress and protecting neurons, thanks to flavon Chrysin (**Figure 4c**), able to determine a neuroprotective microglia M2 polarization via TREM2. Docking experiments supported the ability of the flavon to bind the protein (129).

Albeit the progress concerning the characterization of new TREM-2 ligands, the involvement of this receptor in neurodegenerative disease made necessary the development of new strategies of ligand-screening and synthesis. In this regard, the promiscuous character of this receptor also explains the fact that different ligands, with greater or lesser avidity, were able to activate different signalling pathways leading to different cellular responses (130), with subsequent and respective activation or deactivation of the cell functions for which this protein was responsible.

###### 2.1.2.1.3 TREM-3 receptor

TREM-3 is an activating receptor presenting strong similarity to TREM-1 and TREM-2, regarding DAP12 mediated signalling pathway. TREM-3 is mainly expressed on macrophage, but low level of protein transcripts could also be detected in mouse T cell lines. However, in humans, TREM-3 is a pseudogene (58).

Although little is known about TREM-3, recent findings suggest that it may play a role in modulating TLR signalling. In macrophages, TREM-3 transcripts were upregulated by LPS (131), IL-1β and TNFα (65), and down-regulated by IFN-gamma (131). These observations were confirmed in TLR4 mutant mice, where LPS injection failed to alter the expression of TREM-1 and TREM-3, indicating that this response is dependent on TLR4 signalling (65). Evidence also suggest that TREM-3 may contribute to the inflammatory response of hepatic macrophages and endothelial cells during acute endotoxemia (65), and may play a protective role in host defence against *Klebsiella pneumoniae* infection *in vivo* (132).

However, the discovery of possible ligands that bind to TREM-3 could help the understanding not only of its physiological role but also highlight the insights of the TREM receptors regulation system.

#### 2.1.3 TREM-like receptors

TREM-like receptors, TREM-like-1 (TREML-1), TREML-2 and TREML-3, are encoded within the TREMs gene cluster in humans. It must be pointed out that the TREM-like proteins have distinct structural and functional properties compared to TREM-1 and TREM-2 (133). Particularly, TREML-1 can enhance the calcium signalling in an SHP2 (PTPN11)-dependent manner, whereas some genetic variant of TREML-2 have been identified to be protective for Alzheimer’s disease, contrary to TREM-2 genetic variants. The role of TREML-3 remains to be investigated (133).

TREML-1 is widely expressed in microglia and its levels in the brain have been associated with decreased risk of developing Alzheimer disease in humans (134). This receptor shows similar binding affinity with Aβ oligomers as TREM-2 (114). TREM-2 knockout showed a dramatic increase of TREML-1 expression in the brain (135). Interestingly, the expression of an alternative transcript of TREML-1 (TREM like-1s) has been found to interact with TREM-2 *via* an immunoprecipitation assay and its over-expression inhibited osteoclast differentiation of bone marrow-derived macrophages (136). Therefore, TREML-1s could be a negative regulator of TREM-2 function.

These data suggest a plausible role of TREML-1 as TREM-2 antagonist, in both physiological and pathological conditions. Moreover, finding new molecules that can act *via* TREML-1 could modulate the activity of TREM-2, and *vice-versa* On the other hand, also targeting TREML-2 could open to new strategies for the treatment of Alzheimer’s disease, highly enhancing the rate of success. Considering its protective role, an activating molecule could represent a possible preventive treatment for individuals with specific genetic variations already known to greatly increase the risk of Alzheimer’s disease.

#### 2.1.4 SIRP family members

SIRP (also called signal regulatory proteins) are mainly present in myeloid cells. There are three main SIRP receptors: SIRP-α, SIRP-β and SIRP-γ. All SIRPs members present an extracellular region that consists of three immunoglobulin superfamily (IgSF) domains and have different cytoplasmic regions (137). SIRP-α does contain an ITIM motifs, which mediate its association with the phosphatase SHP2 (138). SIRP-β has a very short cytoplasmic region of only 6 amino acids which binds to the adaptor protein DAP12 and transmit activating signals (139). Whereas SIRP-γ has a shorter cytoplasmic region (of only 4 amino acids), lacking a charged amino-acid side chain that allows the association with DAP12 (137, 140). Recently, it was shown that SIRP-β1 acts as a phagocytic receptor on microglia in amyloid precursor protein transgenic mice and in patients with Alzheimer’s disease. Indeed, activation of SIRP-β1 on cultured microglia induced reorganization of the cytoskeleton protein β-actin, increased phagocytosis of fibrillary amyloid-β (Aβ) (**Figure 3**), and suppressed LPS-induced gene transcription of TNF-α and nitric oxide synthase-2 (139).

The first ligand identified for SIRP-α was CD47 (141), an immune checkpoint molecule that downregulates key aspects of both the innate and adaptive antitumor immune response. CD47 is also a ligand for SIRP-γ (10 times weaker), but not for SIRP-β (142). An automated quantitative TR-FRET-based high-throughput screening assay platform reports the screening of large diverse drug-like chemical libraries to discover novel small molecules able to inhibit CD47-SIRP-α interaction (143). Five compounds were identified, whose structural identification is still under investigation (143). SIRP-α has also been shown to bind to the surfactant proteins A and D (SP-A and SP-D), the first at high levels in the lungs, the second in all mucosal surfaces, but with less affinity than CD47 (144). Most likely, SIRP-α recognize the globular heads glycosylated groups of surfactant proteins SP-A and SP-D. It has been proposed that SP-A and -D present a dual function, both helping to maintain an anti-inflammatory response by stimulating SIRP-α during the interaction with phagocytosis-stimulating PAMPS and inducing a pro-inflammatory response (144).

Stimulation by SIRP-β monoclonal antibody triggers SYK phosphorylation, MAPK activation, phagocytosis on macrophages (140) and migration of neutrophils (145). The natural ligand of SIRP-β is unknown and yet its biological significance remains unresolved.

Finally, several interesting questions for SIRP receptors remain to be answered, especially linked to their extracellular and intracellular ligands, and the research of new binding molecules could be extremely helpful in cancer and inflammation field.

#### 2.1.5 Siglecs family members

Siglecs (sialic acid binding immunoglobulin-like lectins) are I-type (Ig-type) lectins characterized by an Ig domain that mediates sialic acid binding. Sialic acids are a large family of 9-carbon sugars that are all derivatives of neuraminic acid (Neu) or keto-deoxynonulosonic acid (KDN) (**Figure 4d**) (146). They are typically added at the end of the glycosylation process, capping a diverse array of glycosylation structures (146). Therefore, they are present on a variety of proteins and lipids attached to a wide of mammalian cell surfaces, including all human cells (147), playing key roles in Siglecs-mediated immune regulation and maintaining self-tolerance. Siglecs are a family of 14 different receptors which can be divided into two main groups based on their genetic similarity. The first group is present in all mammals consisting of Siglec-1 (Sialoadhesin), Siglec-2 (CD22), Siglec-4 (MAG), and Siglec-15 (148). The second group includes the CD33-related Siglecs, named Siglec-3 (also called CD33), −5, −6, −7, −8, −9, −10, −11, −14, and −16 (149). They are mainly expressed on monocytes, monocyte-derived macrophages and monocyte-derived DCs but also in B cells, basophils, neutrophils, and NK cells, with different expression patterns for every cell subset (150).

Each Siglec presents a specific role in the regulation of immune cell function in infectious diseases (151), inflammation, neurodegeneration, autoimmune diseases and cancer (152). Siglec-8 is involved in the development of asthma and Siglec-9 chronic lung inflammation (151). Whereas, Siglec-3, with ITIM motif, inhibits microglial uptake of amyloid beta and genetic variation could increase the development of Alzheimer’s disease susceptibility (153–155). Collectively the data report that Siglec-3 can inhibit the clearance of amyloid plaque in microglial cell cultures and *in vivo* (155), considering that its knockdown could mitigate amyloid-β pathology (**Figure 3**) (154). Interestingly, both Siglec-3 and TREM-2 have been associated with increasing the risk of Alzheimer’s disease, but in an opposite way. Indeed, conversely to Siglec-3, TREM-2 reduces plaque load and upregulates phagocytosis genes (**Figure 3**) (156). The Alzheimer-linked genetic R47H variant of TREM-2 acts similarly to Siglec-3, impairing amyloid-β–induced microgliosis and microglial activation (157). The link between TREM-2 and Siglec-3 has been straightened by knockdown experiments, which have shown that TREM-2 acts downstream of Siglec-3 and that loss of microglial clearance capacity might be reversed by therapeutic inhibition of Siglec-3 or activation of TREM-2 (158). Therefore, targeting those receptors could facilitate therapeutics to treat neurodegenerative pathologies as Alzheimer’s disease.

Recently, curcumin has been proposed as an immunomodulatory treatment capable of emulating anti-β-amyloid vaccine in stimulating phagocytic clearance by reducing Siglec-3 and increasing TREM2 (159). However, even if curcumin does not represent a new ligand for neither TREM2 or Siglec-3, these findings suggest the huge potential that could derive by controlling both TREM2 or Siglec-3 pathways involved in developing Alzheimer’s disease (**Figure 3**).

The scientific interest has been so far focused on finding Siglec-3 inhibitors that might be effective against disease progression. Microparticles of subtype-selective trisaccharide containing 2,5,9-trisubstituted sialic acid mimetic, called P22, increases the uptake of the toxic Alzheimer’s disease peptide and amyloid-β into microglial cells (160), evidencing Siglec-3 as promising target for therapeutics favouring clearance of amyloid-β.

Siglec-4 and Siglec-14 till Siglec-16 do not have ITIM or ITIM like motifs. Their signals are mediated by DAP12, and, therefore, called activating Siglecs (161). Upon ligand binding, Siglecs-DAP12 system can recruit PI3K thus promoting an inflammatory response through activation of MAPK pathway (162). Other Siglecs, and in particular CD33-related Siglecs, have an ITIM and/or ITIM-like motif in their intracellular domain and can mediate an inhibitory signal (151).

As described above, the presence of sialic acids structures on cell surface could function as a self-associated molecular pattern (SAMP) and thus, Siglecs can act to dampen leukocyte activation under homeostatic conditions (162, 163).

Interestingly, several pathogenic bacteria also use the sialic acid-Siglec axis to dampen the immune system in favour of their survival. In this regard, they could have acquired the ability to take sialic acids or sialylated structures from the host, to synthetize “mimic” structures or even perform *de novo* synthesis of sialic acids, giving them a survival advantage (161). The same strategy is also used in case for the Human immunodeficiency virus (HIV) and the Porcine reproductive and respiratory syndrome virus (PRRSV) (164, 165). It has been demonstrated an increased sialylation, α2,3; α2,6, and α2,8 linked sialic acids, in multiple tumour tissues like renal cell carcinoma, prostate cancer, colon cancer, breast cancer, head and neck squamous cell carcinoma and oral cancer (166).

Finally, Siglecs participate in the discrimination between self and non-self sialic acid motifs, triggering endocytosis, pathogen and dysfunction recognition and regulating, both by activation and deactivation, the function of innate and adaptive immune cells. The research, finding and develop of sialyl-based small chemical entities could pave the way for new therapeutic treatments in different pathological conditions like infectious diseases, inflammation, neurodegeneration, autoimmune diseases and cancer.

### 2.2 C-type lectin receptors

C-type lectin receptors (CLRs) is a family of receptors which possess one or more carbohydrate-recognition domains, thanks to which they usually bind carbohydrate moieties through a Ca^2+^ dependent conserved motif, although some of them lack the Ca^2+^ binding site. These proteins differ in the kind of pathogen-derived or self-expressed ligands that they are able to recognize. Among these ligands, there are not only glycans but also proteins or lipids, triggering functions as adhesion, phagocytic, and signalling pathways and directing the cell towards homeostasis following the activation of innate and adaptive immunity. CLRs represent a huge group of proteins divided into seventeen subgroups (167). Considering the ability of these proteins to recognize polymeric structures, some members of this family are able to also interact with small structural motifs, as DC-Specific Intercellular adhesion molecule-3-Grabbing Non-integrin (DC-SIGN) and Macrophage-inducible C-type lectin (Mincle) (**Table 2**), two members particularly studied and crucial in the immune response modulation.

**Table 2:** Overview of DC-SIGN and Mincle ligands.

Receptor	Ligand	Biological function	Reference
DC-SIGN	Carbohydrate structures (high mannose or fucose) on the surface of pathogens like:		
	a) HIV	Transmission of viral pathogen transport to lymphoid tissues	(172)
	b) CMV	Transmission of infectious viral particles to different permissive cells and enhancement of infection and CMV replication in DC-SIGN-expressing THP-1 cells	(173)
	c) Ebola	Binding to Ebola virus and transmission of the infection to susceptible cells	(174)
	d) Dengue	Binding to four serotypes of Dengue and transmission of the infection to susceptible cells	(175)
	e) hepatitis C	Capture and delivery of the hepatitis C virus to the liver and modulation of dendritic cell function	(176, 177)
	f) Leishmania	Leishmania uptake by dendritic cells and immunoregulation of cutaneous leishmaniasis	(178)
	g) *Candida albicans*	Internalization of C. albicans in specific DC‐SIGN‐enriched vesicles	(179)
	Galactofuranose	Secretion of the pro-inflammatory cytokines IL-6 and TNF-α	(184)
	Lipoglycan lipoarabinomannan of the *Mycobacterium Tubercolosis*	Cellular immune response suppression of both immature DCs and mature DCs	(180–182)
	Mannone lipoglycan lipoarabinomannan of *Mycobacterium bovis*	Impairment of LPS-induced maturation of dendritic cells and increase of immunosuppressive cytokine IL-10 production	(188)
	Glycolipids and glycosphingolipid from *Schistosoma mansoni cercariae*	–	(183)
Mincle	Trehalose 6,6′-dimycolate	Immunostimulatory activity	(203)
	Trehalose dibehenate	Activation Syk-Card9 signaling in APCs; Th1/Th17 response	(204)
	Glyceroglycolipid and a mannosyl fatty acids linked to mannitol from *Malassezia* sp*ecies* extracts	–	(206)
	Glycolipids from *Mycobacterium tuberculosis*	Activation of the Syk-Card9 signaling pathway in macrophages	(207)
	Cholesterol crystals	Production of pro-inflammatory cytokines	(210)
	β-glucosylceramide	Immunostimulatory activity in myeloid cells	(212)
	Cholesterol sulfate	Production of proinflammatory mediators, severe local inflammatory response	(194)
	Desmosterol	–	(211)
	Sitosterol	–	(211)

#### 2.2.1 DC-SIGN

DC-SIGN receptor (or DC specific Intercellular adhesion molecule-3-Grabbing Non-integrin) is expressed by myeloid DCs and subpopulations of macrophages (168). DC-SIGN is highly expressed, and considered as marker, in cancer-associated fibroblasts and M2 macrophages, which are involved in the malignancy of different tumours (169). It has been reported that the expression of DC-SIGN in serum and cancer tissues may affect the survival time for colon cancer patients, representing also a valuable target for cancer treatment (170). Even though, DC-SIGN can induce IL-10 pathways, such as those including ERK and PI3K (171), whether the receptor is relevant to the induction of diseases like asthma remains to be determined.

DC-SIGN recognizes specific carbohydrate structures (high mannose or fucose) on the surface of pathogens and self-glycoproteins. This protein is particularly known to be the receptor that captures HIV-1 at sites of entry, enabling its transport to lymphoid tissues, where DC-SIGN efficiently transmits low amounts of HIV-1 (172).

Besides HIV-1, DC-SIGN was also shown to bind other viruses like cytomegalovirus (173), Ebola (174), Dengue (175), and hepatitis C (176, 177), as well as microorganisms as *Leishmania* (178) and *Candida albicans* (179). The protein is able to bind *Mycobacterium tuberculosis* and thus mediating its entry in DCs *in vivo*. In this scenario, it does not only allow *Mycobacterium Tubercolosis* to infect DCs but also suppresses the cellular immune responses of both immature DCs and mature DCs (180). Indeed, DC-SIGN specifically binds to the lipoglycan lipoarabinomannan (LAM), a major component of the mycobacterial cell wall, which contains a carbohydrate backbone composed of D-mannan and D-arabinan (181, 182). Interestingly, DC-SIGN does not bind to all mycobacterium similarly, suggesting that it may act through selective recognition. In fact, *Mycobacterium smegmatis*-derived LAM, capped by phosphoinositide residues (PILAM), or *Mycobacterium fortuitum* and *Mycobacterium chelonae*-derived AraLAM, Arabian domain uncapped, does not bind to DC-SIGN. Also, *Mycobacterium avium* derived ManLAM, capped with single mannose residues, was also poorly recognized by DC-SIGN (181). All the data suggest that this receptor recognizes a specific motif domain. DC-SIGN is also able to bind small chemical entities like glycolipids derived from *Schistosoma Mansoni cercariae* and their excretory or secretory such as carbohydrate moieties of both glycosphingolipid species with Galβ1–4(Fucα1–3)GlcNAc (LewisX) and Fucα1–3Galβ1–4(Fucα1–3)GlcNAc (pseudo-LewisY) determinants (183). Recently, it was shown that Galactofuranose (Galf) can interact with DC-SIGN and induce the secretion of the pro-inflammatory cytokines IL-6 and TNF-α (184).

Interestingly, DC-SIGN is organized in plasma membrane microdomains (with average diameter of 200nm) crucial for binding and internalization of virus particles, acting as a docking site for pathogens (185). It was also suggested that cholesterol-dependent membrane properties, rather than lipid rafts *per se*, are responsible to promote efficient HIV-1 infection in T cells (186).

DC-SIGN is also an essential co-receptor for TLR4-induced activation of human DCs. Indeed, fucosylated glycan, upon binding with DC-SIGN in DCs, primed naïve T cells towards a Th1 profile, while inducing TLR4-activation (187). Interestingly, upon *mycobacterium bovis* ManLAM binding, DC-SIGN impairs LPS-induced maturation of DCs and increases the production of the immunosuppressive cytokine IL-10, highlighting a possible pathogen strategy to escape immune surveillance (188). Notably, this receptor, encountering and recognizing a pathogen, is able to stimulate the kinase Raf-1, inducing acetylation of the NF-κB subunit p65 and increasing anti-inflammatory cytokines expression, all this only after TLRs-induced activation of NF-κB. This evidence shows a receptor ability to induce adaptive immunity by DCs against pathogenic microorganisms (189).

Considering DC-SIGN specific ligand mediated immune response, polyvalent carbohydrate ligands have been proposed in the design of novel immunomodulants and vaccine adjuvants (190). Finally, DC-SIGN is an important player in the recognition of pathogens by dendritic cells and uncover more functional aspects of its activation mechanisms will be extremely important for the treatment of life-threatening infection disease, such as Dengue or Ebola, also considering that this protein seems to be able to recognize small structural motifs as well as polymeric structures. Moreover, new molecules that bind with more affinity to DC-SIGN could be used as antagonists to prevent the binding of other pathogens or as vaccine adjuvants.

#### 2.2.2 Mincle

Mincle (also called macrophage-inducible C-type lectin) is mainly expressed on monocytes, macrophages, neutrophils and DCs (191). However, in macrophages and DCs, its expression can be strongly upregulated by PAMPs, such as the TLR4 ligand LPS or by a Mincle ligand (192). Mincle has been reported to be associated with rheumatoid arthritis (193), but also to other inflammatory-mediated diseases. Indeed, this receptor is also involved in allergic skin inflammation (194) and post-ischemic inflammation (195), and other experimental inflammatory models such as chronic alcoholic liver disease (196). Given the roles that Mincle plays in a (chronic) immune reaction, it has been suggested both as adjuvant for the treatment of pathogenic immune of Crohn’s disease (197) and also for foot-and-mouth disease virus infection (198). Recent studies show that Mincle also contributes to neuropathic pain in the dorsal root ganglia and spinal dorsal horn (199). Furthermore, Mincle promotes and maintains inflammatory phenotypes of M1 macrophages in acute renal inflammation (200).

The protein is an activating receptor coupled with the ITAM–bearing adaptor FcRγ chain (201). Its expression is sensitive to various inflammatory stimuli, such as LPS, TNF-α, IL-6, and IFN-gamma, activated downstream of the nuclear transcription factor NF-IL6 in macrophages (202).

**Figure 5:**
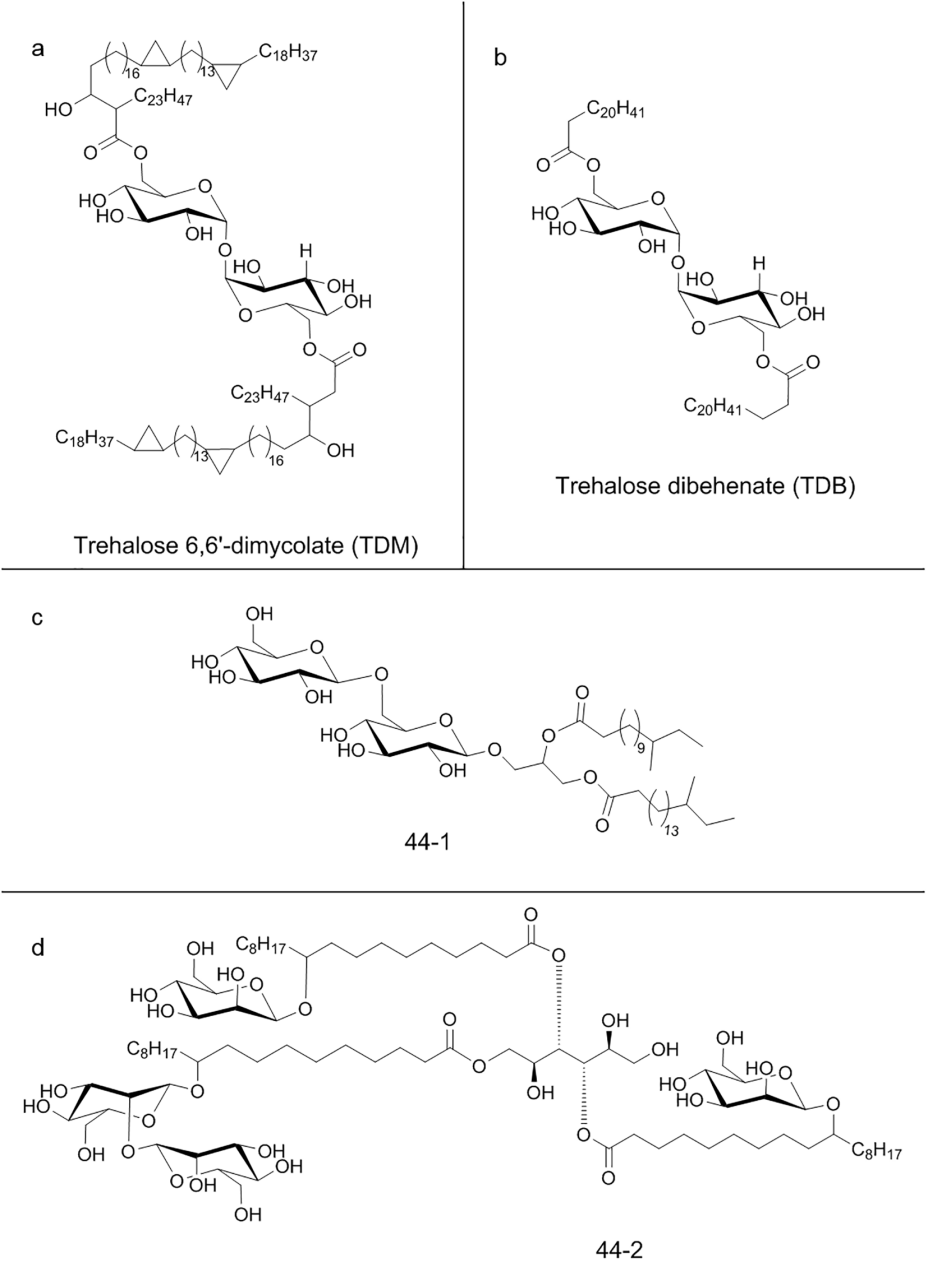
Chemical structure of some C-type lectin receptors ligands: **(a)** Trehalose 6,6′-dimycolate (TDM); **(b)** Trehalose dibehenate (TDB); **(c)** Glucosyl-based glyceroglycolipid (44-1); **(d)** Mannosylated fatty acids linked to mannitol (44-2).

Mincle specific ligand is trehalose 6,6′-dimycolate (TDM) (**Figure 5a**), a mycobacterial cell wall glycolipid and its most studied immunostimulatory component (203). Mincle is also able to recognize analogs like trehalose dibehenate (TDB) (**Figure 5b**) but not the non-glycosylated mycolate or trehalose molecules (204). Though TDM is also present into cell wall of other mycobacteria, corynebacteria, nocardia and fungi, Mincle is not able to bind all of them, suggesting more specific and complex recognition mechanisms. Also, the Mincle ligand in *Malassezia* sp*ecies* fungi has not yet been identified, suggesting the existence of ligands rather than TDM (205). Ishikawa et al. were able to identify two ligands after fractionation of *Malassezia* extracts: a glucosyl-based glyceroglycolipid (named 44-1) (**Figure 5c**) and a unique mannosylated fatty acids linked to mannitol (named 44-2) (**Figure 5d**) (206). Moreover, Mincle is also able to recognize glycolipids of *Mycobacterium tuberculosis* (207). Recently, it has been designed an alkyl 6-*O*-acyl-β-D-glucosides that resulted to be an effective agonist of Mincle, with a potency comparable to the prototypical ligand TDM (208).

Furthermore, it was found that *L. prolificans* and *S. boydii* α-glucan polysaccharides were recognized by Mincle through the α-(1→4) and α-(1→6)-linked glucopyranoside moiety, and Mincle deficiency impacts the phagocytosis dynamics (209).

Notably, Mincle recognizes cholesterol crystals producing pro-inflammatory cytokines (210) and cholesterol sulfate of the epithelial layer inducing local secretion of different proinflammatory mediators (194). The receptor can also signal through other sterols including, desmosterol and sitosterol (211). Mincle has further crucial function in regulating the immune system, being capable to sense and react to tissue damage. Indeed, β-glucosylceramide, identified as endogenous ligand for Mincle upon cell damage, enables an immunostimulatory activity in myeloid cells and the induction of inflammatory cytokine production (212).

Block of TLR4 or NF-κB suppressed LPS-induced Mincle expression in macrophages (**Figure 3**) and maintained M1 phenotype through Syk pathway (213). Furthermore, Mincle acts in synergism with TLR7/8 by inducing NF-κB signalling in monocyte-derived DC, thereby enabling the production of Th1-polarizing cytokines and promoting autologous Th1 polarization (200).

Although Mincle shows binding ability to different classes of ligands influencing the innate immune system, such as lipids and cholesterol derivatives (**Figure 1b**), there is a broad spectrum of potential further agonists for Mincle, with some ligands already exhibiting potential as vaccine adjuvants. Nevertheless, much remains to be unanswered in terms of better understanding the pathways involved in Mincle activation upon ligand interaction.

## 3 Conclusions

The innate immune system senses microbial infection, cell dysfunction and self-ligands by pathogen recognition receptors (PRRs), such as TLRs and regulatory receptors, usually associated with different ITAM-bearing signalling units, as FcRγ and DAP12. The rapid activation of PRRs signalling must be followed by counterbalanced regulatory inhibitory action, aimed to make the immune reaction effective and non-harmful, so preventing cytotoxic effects related to excessive activation. Recently immunoglobulin-like and C-type lectin receptors have gained interest as targets for vaccine development and immune therapies for their ability to activate and finely modulate the immunological functions. These processes are mediated by endogenous and exogenous molecules, most of which are not yet known, often structurally characterized by small size and lipidic nature.

For this reason, small chemical entities represent a target for the characterization of biological mechanisms determining receptor-mediated cell functions along with the development of specific receptor-associated disorders therapies. The main topic of this review is, in fact, the description of the main immunoglobulin receptors and CLRs, seen not only from a biological and functional point of view, but also considering their binding with small associated specific ligands. In this regard, LMIR, TREM, Siglec, SIRP and the most studied CLRs receptors were analysed for their ability to recognize and functionally act upon small molecules interactions.

Different classes of molecules such as phospholipids, glycolipids and sulfolipids can bind to several immunoglobulin-like receptors, like LMIRs and TREMs, characterized by activating/tuning/inhibitory roles. The sharing of ligand families suggests strong evidence of a complex mechanism of regulation though TREMs-LMIRs-axis. This cellular dynamic represents a finely tuned immunomodulatory mechanism, that needs further investigation to better understand the fine level of complexity of these receptors in response to endogenous and/or external stimuli (43). Indeed, the signalling pathway activated upon binding of the two receptors families could synergise or even bifurcate their signalling pathways in opposite biological effects, because of different cellular and physiological states that can potentially cause expression of different ligand molecular entities. Furthermore, considering that Siglec and SIRP receptors share similar downstream pathophysiological effects as TREM family members, and since little is known about their endogenous ligands, a more detailed study of a possible synergism between these receptors should be highlighted. In addition, Siglec and SIRP are involved in the antitumor immune response, so further studies on their possible binding partners could be extremely useful in both cancer and inflammatory research. Several common features were evident also among CLRs sub-families. In this regard, yeast mannans appear to possess same immunostimulatory properties, which are selectively recognized by DC-SIGN and Mincle, also capable of mediating an immunomodulatory activity thanks to the interaction with small glycoconjugates (107).

Interestingly, a close similarity or structural equality of some ligands can also be highlighted for some immunoglobulin-like receptors and CLRs. In particular, glycolipids and sulfolipids, but also ceramide compounds, have shown binding affinities for LMIRs, TREM-2 and Mincle receptors. However, there are other common feature among this class of receptors. As previously described, despite the structural similarity among the immunoglobulin like receptor family, there are relevant difference between activating and inhibitory receptors. Similarly, Mincle but also other CLRs, behave more like the activating immunoglobulin-like receptors, which engage kinases indirectly, through association with ITAM-bearing signalling chains of DAP-12 or FcRγ (13, 214). Thus, although our review highlights the classification into two main classes of receptors, they may have distinct signalling requirements based on activation by distinct or similar ligands, which could lead to subtle differences in their downstream responses.

Interestingly, TLRs act in synergism with most of the receptors described. Indeed, the TLR/CLR antagonist combinations is one of the most well know adjuvation systems to enable early-life immunization against intracellular pathogens (200), already investigated for Dectin-1, Dectin-2, DC-SIGN and Mincle (190, 197, 215–217). To this end, it would be very interesting to identify new ‘adjuvant’ molecules based on other receptors described in this review, such as receptors belonging to the immunoglobulin like receptors family, and thus amplify the opportunity to enhance strategies for patient-immunization.

Currently, the exact structure of many ligands that interact with those receptors is yet not fully characterized and further investigation must be performed to better understand the functional mechanism underlying the receptor activation. In general, small molecules offer a unique advantage to maximize the therapeutic benefits, especially targeting membrane protein, with potential low production costs and huge possibility to modulate and optimize activity by chemical synthesis and structural modification. Therefore, deciphering the biological activity of these ligands, together with medicinal chemistry and molecular biology, could lead to the identification and optimization of novel small molecules able to inhibit or enhance the function of the receptor of interest. However, there are also disadvantages, as small molecule ligand could reveal sub-micromolar affinities for several targets besides the target of interests (218). Therefore, conducting more preclinical studies and enhancing efforts before the clinical phases are of extreme importance. This approach will help to develop more effective drugs and reduce financial consequences of failure (219). A fine study of each receptor family could at least reduce unwanted *in vivo* side effect and interchangeable ligand-receptor affinity.

Finally, mastering the binding specificity of each receptor could advance disease understanding and improve the evaluation of biomarker-guided treatment strategies.
